# Stability of child appetitive traits and association with diet quality at 5 years and 9–11 years old: Findings from the ROLO longitudinal birth cohort study

**DOI:** 10.1038/s41430-024-01436-6

**Published:** 2024-04-04

**Authors:** Anna Delahunt, Sarah Louise Killeen, Eileen C. O’Brien, Aisling A. Geraghty, Sharleen L. O’Reilly, Ciara M. McDonnell, Rosemary Cushion, John Mehegan, Fionnuala M. McAuliffe

**Affiliations:** 1 UCD Perinatal Research Centre, School of Medicine, University College Dublin, National Maternity Hospital, Dublin 2, Ireland; 2 School of Biological, Health and Sport Science, Technological University Dublin, Dublin, Ireland; 3 School of Agriculture and Food Science, University College Dublin, Dublin, 4 Ireland; 4 Department of Paediatric Endocrinology & Diabetes, Children’s Health Ireland Temple St & Tallaght, Dublin, Ireland; 5 Trinity Research in Childhood Centre (TRICC), Discipline of Paediatrics, School of Medicine, Trinity College, Dublin, Ireland; 6 School of Public Health, Physiotherapy and Sport Science, University College Dublin, Dublin, Ireland

**Keywords:** Nutrition, Paediatrics, Health care

## Abstract

**Background:**

We explored change in child appetitive traits from 5 to 9–11 years old and examined associations between appetitive traits at both timepoints and child diet quality.

**Methods:**

This is secondary analyses of the ROLO longitudinal birth cohort study, including mother-child dyads from the 5 and 9–11-year old follow-up. The Children’s Eating Behaviour Questionnaire measured child appetitive traits, with 167 children having matched data for both timepoints. The Healthy Eating Index (HEI) measured diet quality. Linear mixed models and multiple linear regression were completed.

**Results:**

Mean (SD) score for ‘Emotional Overeating’ (1.63 (0.51) vs. 1.99 (0.57), *p* = <0.001) and ‘Enjoyment of Food’ (3.79 (0.72) vs. 3.98 (0.66), *p* = <0.001) increased from 5 to 9–11 years. Mean score for ‘Desire to Drink’ (2.63 (0.94) vs. 2.45 (0.85), *p* = 0.01), ‘Satiety Responsiveness (3.07 (0.66) vs. 2.71 (0.66), *p* = <0.001), ‘Slowness Eating’ (3.02 (0.77) vs. 2.64 (0.78), *p* = <0.001), and ‘Food Fussiness’ (3.00 (1.04) vs. 2.81 (0.96), *p* = 0.001) decreased. At 5-years-old, ‘Food Responsiveness’ and ‘Enjoyment of Food’ were positively associated with HEI and ‘Desire to Drink’, ‘Satiety Responsiveness’ and ‘Food Fussiness’ were negatively associated with HEI. At 9–11-years, ‘Enjoyment of Food’ was positively and ‘Desire to Drink’ and ‘Food ‘Fussiness’ were negatively associated with HEI.

**Conclusions:**

Food approach appetitive traits increased over time, whereas food avoidant appetitive traits tended to decrease. At both time points ‘Food Fussiness’ and ‘Desire to Drink” were inversely associated with HEI. Further research on how appetitive traits track over childhood and how this relates to dietary quality and weight is warranted.

## Introduction

Childhood appetitive traits influence patterns of energy intake and food preference and have been linked to both excess adiposity and poor weight status [1–3]. Previous research has demonstrated continuity in appetitive traits over childhood, in that traits demonstrated in early childhood strongly correlated with those later in childhood [4]. Longitudinal research has observed that, children showed distinct patterns of eating across childhood, such as emotional overeating or heightened food responsiveness, suggesting that the way an individual interacts with their food environment persists over time [5].

Although children appear to retain traits over time, small to medium changes in the degree to which an appetitive trait manifests have been shown to occur throughout childhood [6]. The evidence suggests that appetitive traits related to excess adiposity tend to increase with age [4]. Appetitive traits such as ‘Food Responsiveness’, ‘Enjoyment of Food’ and ‘Emotional Eating’ have been reported to increase over early childhood [4]. Research demonstrates that childhood excess adiposity tracks across the life course [7, 8] therefore identifying the trajectory of appetitive traits related to overweight and obesity could help identify those at risk of unhealthy weight status. Prevalence figures suggest that appetitive traits related to food avoidance, such as ‘Food Fussiness’, ‘Slowness Eating’ or ‘Satiety Responsiveness’ may diminish overtime [9]. Studies of children with fussy eating have demonstrated that this behaviour reduces from early childhood to late childhood, except for a minority whose food fussiness persists [10].

‘Food approach’ appetitive traits such as ‘Food Responsiveness’, ‘Emotional Eating’ and ‘Enjoyment of Food’ have been linked with increased weight status [2, 11]. However, there is a paucity of research examining child diet quality in relation to these traits. A greater volume of research has explored the relationship between ‘food avoidant’ appetitive traits and dietary patterns. Research shows that the diets of children who display fussy eating may lack variety [12] quality [13] and frequently have low fruit and vegetables intakes [13–15].

Establishing how appetitive traits track from early to late childhood and how these traits influence a child’s diet quality is important when planning interventions or public health programmes related to childhood excess adiposity or problematic food selectivity. This research examines the stability of child appetitive traits from preschool to the preteen stage of childhood and explores how appetitive traits are related to diet quality at both timepoints.

## Methods

This is secondary analyses of data from the ROLO (Randomised Control Trial of a low Glycaemic Diet in Pregnancy) longitudinal birth cohort study. The primary study was a randomised control trial (RCT) of a low glycaemic diet in pregnancy to prevent the recurrence of foetal macrosomia (birth weight >4 kg) [16]. Full detailed methodology and findings are published elsewhere [16].

In brief, women on their second pregnancy, who had previously given birth to an infant weighing >4 kg were recruited (*n* = 800). Those included were < 18 weeks gestation, with a singleton pregnancy, had no previous history of gestational diabetes, and were over 18 years old. Women were randomised to either the intervention group which received dietary advice on a low glycaemic diet, or the control group who received routine antenatal care. Results showed no significant difference in birth weight between the control and intervention groups. Women in the intervention group did, however, reduce their gestational weight gain and improve their glycaemic control [16]. The mother and child dyads from the primary study have been followed up at regular timepoints, including when the children were 5 and 9–11 years old. The current analyses included 306 mother-child dyads from the 5 year and 224 from the 9–11-year follow-up. A total of 167 children had matched data on appetitive traits for both timepoints. Ethical approval was provided by ethics committees at the National Maternity Hospital, Dublin, Our Lady’s Children’s Hospital, Crumlin, Dublin, Ireland (Ethics reference number: GEN/279/12) and the office of Research Ethics Committee, University College Dublin (LS-15-06-Geraghty-McAuliffe).

### Anthropometry

At the 5 and 9–11-year follow-up, maternal and child weight was measured using a calibrated stand-on digital weighing scale (SECA 813 GmbH & co. Kg. Hamburg, Germany) to the nearest 0.1 kg. Participants were measured in light clothing without shoes. Standing height was measured, without shoes, with head aligned in the Frankfort plain, using a free-standing stadiometer (SECA 217 GmbH & co. Kg. Hamburg, Germany) and measurements were recorded to the nearest 0.1 cm. Body mass index (BMI) was calculated as kilogram per metre squared (kg/m^2^). Children’s weight, height, and BMI values were converted to age-and sex-specific z-scores according to the 1990 UK reference data using Excel LMS Growth Macro [17, 18]. BMI z-scores were categorised as ‘Underweight’ (BMI z-score <−2.0), ‘Healthy weight’ (BMI z-score >−2.0 and ≤1.0), ‘Overweight’ (BMI z-score >+1 SD) or Obesity (BMI z-score >+2 SD), using the World Health Organisation criteria for children aged 5 to 19 years [19]. All measurements were taken as per study protocol and were carried out by trained researchers.

### Child appetitive traits

At both timepoints, mothers were asked to complete the Children’s Eating Behaviour Questionnaire (CEBQ). The CEBQ [20] is a parent reported, validated psychometric tool, which was developed to capture individual differences in eating styles that may contribute to both underweight and overweight in children [20]. The CEBQ has been shown to have good internal and test-retest reliability and has been validated against observational measures of eating behaviour [5, 21], corresponding well to children’s energy intake. Each item response is graded on a 5-point Likert scale (‘never to always’), with five items within the CEBQ being reverse scored, due to opposite phrasing. Each question relates to one of eight appetitive traits which can be classed as either ‘food approach’ or ‘food avoidant’. The ‘food approach’ domain represents the degree to which a child has a more avid appetite and greater interest in food and includes ‘Food Responsiveness’, ‘Enjoys Food’, ‘Emotional Overeating’ and ‘Desire to Drink’. The ‘food avoidant’ domain represents the degree to which a child has a smaller appetite and is less interested in food and includes ‘Satiety Responsiveness’, ‘Slowness Eating’ ‘Emotional Undereating’ and ‘Food Fussiness’. Each subscale is summed to give a total score, and this is divided by the number of items within the subscale, to give a mean score of the sum. A higher score indicates the child is more likely to express this appetitive trait. A more detailed description of each subscale is presented in a previous publication [22]. Cronbach alpha was completed on each CEBQ subscale item for the 5 and 9–11 year old follow-up. For the 5-year-old follow-up internal reliability coefficients (Cronbach’s α) ranged from 0.695 to 0.928 and in the 9–11 year old follow-up internal reliability coefficients ranged from 0.710 to 0.917, thus all questions were included in the analysis.

### Food frequency questionnaires

To assess children’s dietary intake, mothers completed a food frequency questionnaire (FFQ) on behalf of their child at both timepoints. This measured their habitual intake over the previous year. The FFQ used at the 5 year and 9–11 year follow-up was a 53-item questionnaire based on the Growing up in Ireland (a nationally representative large longitudinal study of children and young people in Ireland), 5-year, caregiver main questionnaire [23]. The FFQ is divided into different categories including breads/pastas, cake/confectionary, dairy, sugar, sweetened beverages, fruit, vegetables, spreads, and meat/fish and asked mothers to report how frequently (e.g. ‘at least once per week’, ‘most days’, ‘once a day’) their child consumed each food product in the past year. For the 5 year follow-up, portion sizes for each item of the FFQ was allocated using the Food Standards Agency food portions sizes book [24]. McCance and Widdowson’s The Composition of Foods Seventh Edition, Integrated dataset [25] was used to assess habitual dietary intakes. For the 9–11 year old follow-up, portion sizes were allocated using the Irish Food Sources Database for Children aged 9–12 years old [26]. McCance and Widdowson, Composition of Foods, Integrated Dataset 2021, [27] was used for nutrient analysis.

### Healthy Eating Index

Children’s dietary quality at 5 and 9–11 years old was assessed using the Healthy Eating Index-2015 (HEI-2015) [28]. The HEI is a measure for assessing whether a set of foods aligns with the Dietary Guidelines for Americans (DGA) [29]. The latest version, the HEI-2015 reflects the 2015–2020 DGA [30]. The HEI uses a density-based methodology (with the exception of fatty acids, which is a ratio of unsaturated to saturated fatty acids), therefore, foods are assessed as per 1000kcals, rather than absolute amounts. The HEI contains 13 components. Nine ‘Adequacy’ components which address the adequacy of the diet in terms of intakes of necessary food groups. This includes total fruits, whole fruits, total vegetables, greens and beans, total protein containing foods, seafood and plant proteins, whole grains, dairy, and fatty acids (ratio of poly- and monounsaturated fatty acids to saturated fatty acids). The other four foods are ‘Moderation’ components, addressing negative elements of dietary intake; refined grains, sodium, added sugars, and saturated fats. The scores are summed to yield a total score ranging from zero to 100, with a higher score indicating greater adherence to the DGA. The following graded approach can be used to help interpret HEI scores; Overall scores of 90 to 100, or component scores that are 90 to 100% of maximum score: A; Overall scores of 80 to 89, or component scores that are 80 to 89% of maximum score: B; Overall scores of 70 to 79, or component scores that are 70 to 79% of maximum score: C; Overall scores of 60 to 69, or component scores that are 60 to 69% of maximum score: D; and Overall scores of 0 to 59, or component scores that are 0 to 59% of maximum score: F [30]. However, the numerical score is more meaningful and grades should only be used alongside the numerical score [30]. The HEI scoring system uses cups as the method of measurement. To convert cups to gram equivalents the food patterns ingredient database [31] was used. Foods from the FFQ’s were mapped to the different food categories used within the HEI. The equivalent values for the HEI component ‘Added Sugars’ were determined slightly differently to the other nine components (which aligned well with Irish dietary intakes). As amounts of ‘Added Sugars’ can vary considerably between US foods and their equivalents on the market in Europe, McCance and Widdowson composition of foods database [27] was used to estimate the amount of sugar added, using maltose and sucrose. Calculation of the HEI was completed using bespoke scripts written in Python programming language.

### Pubertal development

Mothers provided self-reported estimates of their child’s pubertal status using standardised Tanner staging figures (from Stage 1 to 5) relative to their secondary sex characteristics [32]. Boys were assessed by their corresponding stage of pubic hair distribution, while girls were assessed by their corresponding stage of pubic hair distribution and breast development. Preadolescence was defined as Tanner Stage 1 for pubic hair distribution and breast development.

### Early feeding

Breastfeeding exposure was recorded at the 6 months, 2 and 5 year follow-up. At these timepoints, mothers reported if they had ever breastfed. At the 2-year and 5-year follow-up, mothers reported the age (weeks) their infant commenced solid food. A variable was created to indicate if their child had started solid food as per European [33] and national guidelines .

### Statistical analysis

Continuous data were tested for normality using the Kolmogorov–Smirnov test and visual inspection of histograms. Normally distributed variables were reported as mean and standard deviation (SD). Non-parametric variables were reported as median and interquartile range (IQR 25th–75th). Categorical variables were reported as *n* (%). Paired t-tests examined differences between appetitive traits at both timepoints, for those with matched CEBQ data (*n* = 167). To account for missing data, further analysis using linear mixed-effects modelling was completed to examine change in appetitive traits from 5 to 9–11-years old (*n* = 224). Time was used as the predictor, and child sex, breastfeeding exposure, maternal BMI at the 9–11-year-old follow-up and original RCT group were used as the fixed effects and covariate variables.

Pearson’s correlations were completed to examine relationships between child appetitive traits and HEI and total energy intake at 5 and 9–11-years-old. Those with a correlation p-value of <0.05 were further analysed using multiple linear regression. ‘Total energy’ was not evenly distributed and was log^^10^ transformed for correlation and regression analysis. Prior to linear regression analyses, assumptions were tested. Models were adjusted for child age at the timepoint being analysed, child sex, total energy (kcals) intake, breastfeeding exposure, whether complimentary feeding was introduced as per European and national timing recommendations or not and original RCT allocation group. Statistical analyses were completed using IBM Statistical Package for Social Sciences (SPSS) for Windows, version 24.0 (Armonk, NY: IBM, Corp).

## Results

Maternal demographic details at the 9–11-year-old follow-up (*n* = 224) are displayed in Table 1. Child demographic details at age 5 years (*n* = 306) and 9–11 years old (*n* = 224) are displayed in Table 1. Mean child age at the 5-year follow up was 5.2 years old, with 46.5% male. At the 9–11-year-old follow-up, mean age was 10.0 years old, with 49.6% being male. At the 5 year follow-up 24% of children were classified as having overweight (>+1 SD) or obesity (>+2 SD) and at the 9–11 year follow-up, 28% were classified as having overweight (>+1 SD) or obesity (>+2 SD) as per the World Health Organisation criteria for children aged 5 to 19 years [19]. Mean HEI score at 5 years was 62.5%, and median energy intake was 1162 kcals per day. At 9–11 years old mean HEI was 60.1%, with a median energy intake of 1310 kcals per day. At the 9–11-years-old, 90% were pre-pubertal. Demographic details for the cohort of children with matched CEBQ data at both timepoints (*n* = 167) were similar and are presented in Supplementary Table 1.

**Table 1:** ROLO maternal and child demographic characteristics at 5-year and 9–11-year follow-up.

	*n* (%)	Mean (median)	SD (IQR)
**Maternal characteristics at 9–11** **yr old f/up**			
Maternal age (years)	223	43.10	3.87
RCT group (intervention)	118 (52.7)	–	–
Maternal education – 3rd level or above	132 (68.8)	–	–
Maternal ethnicity (white, Irish)	203 (90.6)	–	–
Maternal BMI (kg/m^2^)	208	(25.60)	(23.36,28.97)
Maternal BMI (overweight/obesity) *n* (%)	117 (57.6)	–	–
**Child characteristics at 5** **yrs**			
Child age (years)	306	5.18	0.15
Child sex (male), *n* (%)	141 (46.5)	–	–
Child BMI z-score	293	0.40	0.87
BMI z-score (overweight/obesity) *n* (%)	70 (23.9)	–	–
HEI score (%)	306	62.51	9.29
Total energy intake (kcal)	306	(1161.15)	(934.65,1431.13)
**Child characteristics 9–11** **yrs**			
Child age (years)	224	(9.79)	(9.25,10.25)
Male, *n* (%)	111 (49.6)	–	–
Breastfeeding exposure	139 (73.5)	–	–
Age of solids introduction (weeks)	185	(23.83)	(19.78,26.00)
BMI (kg/m^2^)	211	17.68	2.77
BMI z-score	211	0.31	1.11
BMI z-score (overweight/obesity) *n* (%)	59 (28.0)	–	–
Pubic hair (Tanner Stage I) *n* (%)	168 (89.9)	–	–
Breast development (Tanner Stage I), *n* (%)	84 (81.6)	–	–
HEI score (%)	224	60.14	8.72
Total energy intake (kcal)	224	(1310.01)	(1037.61,1621.76)
**Child appetitive traits at 5** **yrs**			
Food Responsiveness	306	2.49	0.82
Emotional Overeating	306	1.65	0.52
Enjoyment of Food	306	3.73	0.76
Desire to Drink	306	2.67	0.93
Satiety Responsiveness	306	3.06	0.66
Slowness eating	306	3.04	0.78
Emotional Undereating	306	2.70	0.86
Food Fussiness	306	3.08	0.98
**Child appetitive traits at 9–11** **yrs**			
Food Responsiveness	224	2.53	0.84
Emotional Overeating	224	1.97	0.58
Enjoyment of Food	224	3.94	0.70
Desire to Drink	224	2.43	0.84
Satiety Responsiveness	224	2.72	0.68
Slowness eating	224	2.60	0.80
Emotional Undereating	224	2.52	0.69
Food Fussiness	224	2.86	0.99

Results presented as mean (standard deviation) for normally distributed data, median (25th and 75th percentile) for non-parametric data, and *n* (%) for categorical data. Pubertal status defined as being in Stage I (pre-adolescent) of pubic hair distribution for males; Stage I of pubic hair distribution and Stage I of breast development for females according to the 5-stage Tanner criteria. Child appetitive traits (CEBQ) presented as the mean of the sum of each subscale. *ROLO* Randomised control trial of low glycaemic index diet to prevent recurrence of macrosomia, *RCT* Randomised Control Trial, *HEI* Healthy Eating Index, *BMI* Body Mass Index.

In paired t-tests mean (SD) scores for ‘Emotional Overeating’ (1.63 (0.51) vs. 1.99 (0.57), *p* = <0.001) and ‘Enjoyment of Food’ (3.79 (0.72) vs. 3.98 (0.66), *p* = <0.001) increased from 5 years to 9–11 years. Mean scores for ‘Desire to Drink’ (2.63 (0.94) vs. 2.45 (0.85), *p* = 0.01), ‘Satiety Responsiveness’ (3.07 (0.66) vs. 2.71 (0.66), *p* = <0.001), ‘Slowness Eating’ (3.02 (0.77) vs. 2.64 (0.78), *p* = <0.001), and ‘Food Fussiness’ (3.00 (1.04) vs. 2.81 (0.96), *p* = 0.001) decreased from 5 to 9–11 years (Table 2). In linear mixed-effects models, mean scores for ‘Emotional Overeating’ and ‘Enjoyment of Food’ increased over time. Mean scores for ‘Desire to Drink’, ‘Satiety Responsiveness’, ‘Slowness Eating’, ‘Emotional Undereating,’ and ‘Food Fussiness’ decreased over time (Table 3).

**Table 2:** Differences in mean CEBQ subscales scores at 5 years and 9–11 years.

	Age 5 (years)	Age 9–11 (years)			5 years to 9–11-year difference	
CEBQ subscales (*n* = 167)	Mean (SD)	Mean (SD)	*t*-value	*P-value*		Cohen’s d effect size
Food Responsiveness	2.53 (0.84)	2.52 (0.81)	0.23	0.82		0.02
Emotional Overeating	1.63 (0.51)	1.99 (0.57)	−7.55	<0.001		−0.58
Enjoyment of Food	3.79 (0.72)	3.98 (0.66)	−3.62	<0.001		−0.28
Desire to Drink	2.63 (0.94)	2.45 (0.85)	2.58	0.011		0.20
Satiety Responsiveness	3.07 (0.66)	2.71 (0.66)	6.69	<0.001		0.52
Slowness Eating	3.02 (0.77)	2.64 (0.78)	5.58	<0.001		0.43
Emotional Undereating	2.70 (0.87)	2.57 (0.70)	1.93	0.055		0.15
Food Fussiness	3.00 (1.04)	2.81 (0.96)	3.31	0.001		0.26

Paired sample t-tests. Statistically significant (*p* = <0.05); *CEBQ* Child Eating Behaviours Questionnaire.

**Table 3:** Change in child appetitive traits across time from age 5 to 9–11 years old.

	B	95% CI	*P-value*	B	95% CI	*P-value*	B	95% CI	*P-value*	B	95% CI	*P-value*
	Food Responsiveness			Emotional Overeating			Enjoyment of Food			Desire to Drink		
**Time**	0.19	−0.43,0.81	0.54	−1.33	−1.70,−0.96	<0.001	−0.74	−1.16,−0.31	0.001	0.52	0.13,0.92	0.010
**RCT group**	0.19	−0.69,1.08	0.67	0.02	−0.47,0.51	0.92	0.11	−0.60,0.81	0.76	−0.30	−0.90,0.31	0.334
**Breastfed ever**	−0.33	−1.32,0.66	0.51	0.11	−0.48,0.70	0.72	−0.31	−1.17,0.55	0.48	−0.45	−1.18,0.29	0.29
**Maternal BMI**	0.07	−0.03,0.17	0.17	−0.09	−0.42,0.24	0.59	−0.02	−0.10,0.05	0.59	0.002	−0.07,0.07	0.95
**Child sex**	0.25	−0.64,1.15	0.58	0.01	−0.50,0.50	0.98	−0.22	−0.95,0.51	0.56	0.82	0.20,1.44	0.01

	Satiety Responsiveness			Slowness Eating			Emotional Undereating			Food Fussiness		
**Time**	1.62	1.11,2.13	<0.001	1.65	1.14,2.17	<0.001	0.56	0.08,1.09	0.02	1.11	0.43,1.81	0.002
**RCT group**	−0.12	−0.84,060	0.74	0.07	−0.60,0.74	0.84	0.08	−0.60,0.76	0.81	0.71	−0.64,2.07	0.30
**Breastfed ever**	1.04	0.23,1.84	0.01	0.82	0.08,1.57	0.03	0.95	0.19,1.71	0.02	0.18	−1.33,1.69	0.81
**Maternal BMI**	0.07	−0.01,0.15	0.08	0.02	−0.05,0.10	0.53	0.02	−0.06,0.09	0.66	0.06	−0.09,0.21	0.45
**Child sex**	−0.71	−1.44,0.02	0.06	−0.33	−1.00,0.35	0.34	0.21	−0.48,0.90	0.56	1.11	−2.26,2.48	0.11

Linear mixed models. Models adjusted for original RCT allocation group, breastfeeding exposure, maternal BMI at the 9–11-year-old follow-up and child sex. B = Unstandardised Beta = Fixed effects; Time = Random effect; Statically significant (*p* = <0.05).

Results for correlations between child appetitive traits at the 5 and 9–11 years old and HEI are presented in Supplementary Table 2. In adjusted linear regression, at 5-years old ‘Food Responsiveness’ (*B* = 0.02, 95% CI = 0.01,0.03, *p* = <0.001) and ‘Enjoyment of Food’ (*B* = 0.02, 95% CI = 0.01,0.03, *p* = <0.001) were positively associated with HEI and ‘Desire to Drink’ (*B* = −0.01, 95% CI = −0.02,−0.001, *p* = 0.038), ‘Satiety Responsiveness’ (*B* = −0.02, 95% CI = −0.02,−0.01, *p* = <0.001) and ‘Food Fussiness’ (*B* = −0.04, 95% CI = −0.05, −0.02, *p* = <0.001) were negatively associated with HEI. At 9–11-years old, ‘Enjoyment of Food’ (*B* = 0.01, 95% CI = 0.001,0.03, *p* = 0.03) was positively and ‘Desire to Drink’ (*B* = −0.02, 95% CI = −0.03,−0.001, *p* = 0.03) and ‘Food Fussiness’ (*B* = −0.04, 95% CI = −0.06, −0.02, *p* = <0.001) were negatively associated with diet quality (Table 4).

**Table 4:** Associations between child appetitive traits and Healthy Eating Index at 5 years and 9–11 years old.

	*B*	95% CI		*P value*	Adjusted R^2^
		Lower	Upper		
**CEBQ 5 years**					
Food Responsiveness	0.018	0.008	0.028	<0.001	0.028
Enjoys Food	0.023	0.014	0.032	<0.001	0.068
Desire to Drink	−0.012	−0.023	−0.001	0.038	0.023
Satiety Responsiveness	−0.015	−0.023	−0.007	<0.001	0.054
Food Fussiness	−0.035	−0.047	−0.024	<0.001	0.132
**CEBQ 9–11 years**					
Enjoyment of Food	0.013	0.001	0.025	0.031	0.007
Desire to Drink	−0.015	−0.028	−0.001	0.033	0.077
Food Fussiness	−0.040	−0.055	−0.024	<0.001	0.123

Multiple linear regression; Models adjusted for child age at either 5 or 9–11-year follow-up, child sex, breastfeeding exposure, whether complimentary feeding was introduced as per timing recommendations or not, total energy intake (kcals)(log^^10^ transformed) and original RCT allocation group.

## Discussion

In this exploratory research, the ‘food approach’ appetitive traits of ‘Emotional Overeating’ and ‘Enjoyment of Food’ increased over time whereas ‘Desire to Drink’ decreased. In contrast, appetitive traits related to food avoidance; ‘Satiety Responsiveness’, ‘Slowness Eating’ and ‘Food Fussiness’ decreased from 5 to 9–11 years old. ‘Enjoyment of Food’ at both timepoints was positively associated with HEI and at 5 and 9–11 years old ‘Desire to Drink; and ‘Food Fussiness’ were inversely associated with HEI.

Our results showing an increase in ‘food approach’ appetitive traits over time, concur with previous studies [4, 9]. Ashcroft, Semmler [4] observed that from 4 to 11 years old ‘Enjoyment of Food’, ‘Emotional Overeating’, and ‘Food Responsiveness’ increased over time. In a large population-based birth cohort study examining the trajectory of appetitive traits and their relationship with BMI from 1 to 10 years old, three eating patterns were identified: ‘Overeating’, ‘Undereating’, and ‘Fussy Eating’ [9]. ‘Overeating’ was found to be generally low, but increased with time, whereas ‘Undereating’ and ‘Fussy Eating’ varied substantially across the observed timeframe. Our results indicate similar patterns of change and stability [4, 6, 34]. Our observation of an increase in mean scores for ‘food approach’ appetitive traits may relate to the fact that with age, children gain greater autonomy over what they eat and drink, and may be influenced by peers, especially if eating outside the home. Increased ‘Enjoyment of Food’ at the older timepoint may suggest better acceptance of a greater variety of foods, due to increased food exposure over time.

In our cohorts the percentage of children with a BMI in the overweight/obesity range increased from 5 to 9–11 years old. In view of growing evidence that adiposity tracks across the life course [7] and our findings showing a pattern of increasing expression of appetitive traits that are associated with increased adiposity, ensuring a healthy food environment that discourages excessive unhealthy food choices both at home and outside the home is paramount.

Increased scores seen over time for ‘Emotional Overeating’ may pertain to environmental learned behaviour. Higher scores in ‘Emotional Overeating’ relate to greater energy intake than usual during times of stress and negative emotion [20]. In a previous study of the same ROLO cohort at 5 years old, positive associations were observed between maternal ‘Emotional Eating’ and child ‘Emotional Overeating’ [35]. Although some child appetitive traits are thought to be substantially shaped by genetics, others such as ‘Emotional Eating’ have been shown to be strongly influenced by the home environment [36]. A child may mirror what they are observing in the home and their tendency to eat in response to emotions may become more engrained as the child ages. The potential negative implications of this is that ‘Emotional Overeating’ has been implicated in the development of child and adult overweight and obesity risk [37, 38]. Evidence suggests that maternal overweight/obesity is a key risk factor for child overweight/obesity and the underlying determinants are both environmental and genetic [39]. Strategies to prevent the establishment of ‘Emotional Overeating’ and instil healthy eating habits and a healthy food environment in early childhood are vital to reduce later issues with excess weight gain.

The decrease in mean scores observed in ‘food avoidant’ appetitive traits over time reflect reported food fussiness trends. One third of children will exhibit some food fussiness during the first four years of life, but this behaviour diminishes by later childhood with an estimated 4% being persistently fussy [1]. In a cross-sectional study of children aged 2 to 12 years old, ‘Food Fussiness’, ‘Slowness Eating’, and ‘Emotional Undereating’ had higher prevalence in pre-school aged children than older children. The Avon Longitudinal Study of Parents and Children (ALSPAC) found that over half of mothers described their child as food fussy at 15 months [40], but by 10 years old, only 8% displayed persistent fussy eating behaviour [41]. Low weight status [9], poor dietary quality [42], and anxiety and stress at mealtimes and within families [43] are all implications of food fussiness, therefore evidence demonstrating that this eating behaviour is likely to diminish with age, would be welcomed by parents and caregivers.

Our observed decrease in mean scores for ‘Desire to Drink’ is of interest, as to our knowledge this is the first study to examine this subscale across time. ‘Desire to Drink’ has been linked with higher intake of sugar sweetened beverages (SSBs) in children [44] and greater consumption of SSBs is associated with higher child BMI. However, research is conflicted as to whether this subscale is related to excessive weight gain [45]. In clinical practice, young children who drink excessively often present with poor appetites, fussy eating and food refusal at mealtimes. Therefore, it may be of relevance that we observed that both mean scores for ‘Food Fussiness’ and for ‘Desire to Drink’ decreased from 5 to 9–11 years old.

Dietary patterns are an effective method of assessing relationships between nutrition and health because they can account for the interplay between nutrients and foods consumed within the entire diet [46]. In our cohort, at age 5, the mean HEI-2015 score was 62.5% and at 9–11 years old it was slightly lower at 60.1%. This demonstrates that approximately two thirds of calories were derived from foods rich in phytonutrients within the entire diet, with scores closest to 100 aligning most favourably with key DGA dietary recommendations. In comparison to our cohort, data from the USA reported children’s mean national HEI-2015 score as 54.9% [47], slightly lower than our cohort. They also observed that as age increased, total scores decreased with the total mean score being significantly higher for children in the youngest age group (2–5 years) compared with the two older age groups (6–11 and 12–18 years) (60% versus 54% versus 52%, respectively). At 5 years old, we observed that ‘Food Responsiveness’ and ‘Enjoyment of Food’ were positively associated with HEI and at the 9–11 year timepoint, ‘Enjoyment of Food’ remained positively associated with HEI. Interestingly at both timepoints ‘Desire to Drink’ was negatively associated with diet quality score, which suggests that high intakes of beverages may displace intake of foods that could contribute to a higher diet quality. The CEBQ subscale of ‘Desire to Drink’ questions do not specify what drinks are being consumed, however research has shown a link between ‘Desire to Drink’ and higher intake of SSB’s [44]. Previous research has demonstrated that children lack the ability to compensate for energy consumed in drinks, thus leading to a higher overall calorie intake [48]. Assessing type and volume of fluid intake may be an important indicator of decreased quality diet in children with increased weight status or those with selective eating.

At both timepoints ‘Food Fussiness’ was inversely associated with HEI. In view of ‘Food Fussiness’ scores showing a decrease over time, this may suggest that those who persist to be food fussy may also continue to have lower quality diets. At the 5 year time point ‘Food Fussiness’ accounted for 13% of the variance explained in HEI and at 9–11 years old this was 12%. Findings from the ALSPAC study demonstrate that maintaining high dietary variety from age 4–7 years old was inversely associated with problematic behaviours such as food fussiness [40]. In addition, children with higher dietary variety had higher general interest in food, lower desire to drink, and lower satiety responsiveness, than those with low dietary variety. If as our findings suggest, ‘Food Fussiness’ decreases with age, persisting to offer and expose children to a wide variety of foods is important, to maximise diet quality long-term.

Strengths of this research include the availability of data on children’s appetitive traits using a validated questionnaire, in early and late childhood. Data from earlier ROLO follow-up allowed the inclusion of important confounders relating to early feeding. Data from FFQ for both timepoints allowed calculation of the HEI, which provided an effective method of assessing the diet as a whole. The FFQ data also allowed exploration of child energy intake and its inclusion as a potential confounder in regression models. This study is not without limitations. Smaller numbers completed data on child appetitive traits at the 9–11-year-old follow-up, and despite efforts to increase numbers, a smaller sample of children had matched CEBQ data at both time points. As this study was exploratory in design, sample size calculations were not performed. Selection bias may have been present as mothers from the original ROLO pregnancy study were healthy, on their second pregnancy and more than half had achieved third-level education, therefore this sample may not be fully representative of the general population. In addition the limitation of using self-reported questionnaires has the potential for social desirability bias.

## Conclusion

The pattern of changes observed in our cohort suggest that appetitive traits related to increased food intake increase overtime, whereas those related to food avoidance diminish. Further research is warranted to gain a better understanding of how appetitive traits track through childhood and how they relate to diet quality. This could help to provide guidance for intervention strategies for children who present with excess adiposity or problematic food avoidance.

### Supplementary information

Revised Supplementary Tables

## Data Availability

The datasets used and analysed during the current study are not publicly available in line with ethical approval but are available from the corresponding author on reasonable request.

## References

[1] Cardona Cano S, Tiemeier H, Van Hoeken D, Tharner A, Jaddoe VWV, Hofman A (2015). Trajectories of picky eating during childhood: A general population study: Picky Eating Trajectories. Int J Eating Disord.

[2] Carnell S, Wardle J (2008). Appetitive traits and child obesity: measurement, origins and implications for intervention. Proc Nutr Soc.

[3] Syrad H, Johnson L, Wardle J, Llewellyn CH (2016). Appetitive traits and food intake patterns in early life. Am J Clin Nutr.

[4] Ashcroft J, Semmler C, Carnell S, Van Jaarsveld CHM, Wardle J (2008). Continuity and stability of eating behaviour traits in children. Eur J Clin Nutr.

[5] Sleddens EFC, Kremers SP, Thijs C (2008). The Children’s Eating Behaviour Questionnaire: factorial validity and association with Body Mass Index in Dutch children aged 6-7. Int J Behav Nutr Phys Act.

[6] Farrow C, Blissett J (2012). Stability and continuity of parentally reported child eating behaviours and feeding practices from 2 to 5 years of age. Appetite.

[7] Ward ZJ, Long MW, Resch SC, Giles CM, Cradock AL, Gortmaker SL (2017). Simulation of growth trajectories of childhood obesity into adulthood. N Engl J Med.

[8] Guh DP, Zhang W, Bansback N, Amarsi Z, Birmingham CL, Anis AH (2009). The incidence of co-morbidities related to obesity and overweight: A systematic review and meta-analysis. BMC Public Health.

[9] Herle M, De Stavola B, Hubel C, Santos Ferreira DL (2020). Eating behavior trajectories in the first 10 years of life and their relationship with BMI. Int J Obes.

[10] Taylor CM, Hays NP, Emmett PM (2019). Diet at age 10 and 13 years in children identified as picky eaters at age 3 years and in children who are persistent picky eaters in a longitudinal birth cohort study. Nutrients.

[11] Parkinson KN, Drewett RF, Le Couteur AS, Adamson AJ (2010). Do maternal ratings of appetite in infants predict later Child Eating Behaviour Questionnaire scores and body mass index?. Appetite.

[12] Birch LL, Fisher JO (1998). Development of eating behaviors among children and adolescents. Pediatrics.

[13] Oliveira A, Jones L, de Lauzon-Guillain B, Emmett P, Moreira P, Charles MA (2015). Early problematic eating behaviours are associated with lower fruit and vegetable intake and less dietary variety at 4–5 years of age. A prospective analysis of three European birth cohorts. Br J Nutr.

[14] Taylor CM, Northstone K, Wernimont SM, Emmett PM (2016). Macro- and micronutrient intakes in picky eaters: a cause for concern?. Am J Clin Nutr.

[15] de Barse LM, Tiemeier H, Leermakers ETM, Voortman T, Jaddoe VWV, Edelson LR (2015). Longitudinal association between preschool fussy eating and body composition at 6 years of age: The Generation R Study. Int J Behav Nutr Phys Act.

[16] Walsh JM, McGowan CA, Mahony R, Foley ME, McAuliffe FM (2012). Low glycaemic index diet in pregnancy to prevent macrosomia (ROLO study): randomised control trial. BMJ.

[17] Cole TJ, Bellizzi MC, Flegal KM, Dietz WH (2000). Establishing a standard definition for child overweight and obesity worldwide: international survey. Br Med J.

[18] Cole T. LMS Growth- a Microsoft Excel add-in to assess growth references, based on the LMS Method. Version 2.77, 2012. Available from https://www.healthforallchildren.co.uk/.

[19] de Onis M, Onyango AW, Siyam A, Nishida C, Siekmann J (2007). Development of a WHO growth reference for school-aged children and adolescents. Bull World Health Org.

[20] Wardle J, Guthrie CA, Sanderson S, Rappoport L (2001). Development of the children’s eating behaviour questiionaire. J Clin Child Psychol.

[21] Carnell S, Wardle J (2007). Measuring behavioural susceptibility to obesity: Validation of the child eating behaviour questionnaire. Appetite.

[22] Delahunt A, Conway MC, O’Brien EC, Geraghty AA, O’Keeffe LM, O’Reilly SL (2022). Ecological factors and childhood eating behaviours at 5 years of age: findings from the ROLO longitudinal birth cohort study. BMC Pediatr.

[23] Murray A, Williams J, Quail A, Neary M, Thorton M. A summary guide to wave 3 of the Infant Cohort at 5 years of Growing Up in Ireland. Ireland 2015. Available from https://www.growingup.ie/pubs/Summary-Guide-_Infant-Cohort_Wave-3.pdf.

[24] Food Standards Agency. Food portion sizes. 3rd ed. Crawley H, editor. London TSO; 2006.

[25] Public Health England. McCance and Widdowson’s The Composition of Foods, Seventh Summary Edition, and updated Integrated Database. 2015. Available from https://www.gov.uk/government/publications/compostion-of-foods-integrated-database-cofid.

[26] Lyons J, Giltinan M. The Irish Food Portion size database. 1st ed. Dublin, Ireland; 2013. Available from https://www.iuna.net/.

[27] Public Health England. McCance and Widdowson’s Composition of Foods, updated Integrated Database UK; 2021. Available from https://www.gov.uk/government/publications/composition-of-foods-integrated-dataset-cofid.

[28] Kennedy E, Meyers L, Layden W (1996). The 1995 dietary guidelines for Amercians: An overview. J Am Diet Assoc.

[29] Dietary Guidelines Advisory Committee. Dietary guidelines for Americans 2015-2020 In: Agriculture UDo, editor. USA: USA Governement Printing Office; 2015.

[30] Krebs-Smith SM, Pannucci TE, Subar AF, Kirkpatrick SI, Lerman JL, Tooze JA (2018). Update of the Healthy Eating Index: HEI-2015. J Acad Nutr Dietet.

[31] US Agricultural Research Service. Food Survey’s Reserach Group; Food Patterns Equivalents Database Maryland, USA: US Agriculture Research Service; 2022 [Available from: https://www.ars.usda.gov/northeast-area/beltsville-md-bhnrc/beltsville-human-nutrition-research-center/food-surveys-research-group/docs/fped-databases/.

[32] Tanner JM, Whitehouse RH (1976). Clinical longitudinal standards for height, weight, height velocity, weight velocity, and stages of puberty. Arch Dis Childhood.

[33] Fewtrell M, Bronsky J, Campoy C, Domellöf M, Embleton N, Fidler Mis N (2017). Complementary Feeding: A Position Paper by the European Society for Paediatric Gastroenterology, Hepatology, and Nutrition (ESPGHAN) Committee on Nutrition. J Pediatr Gastroenterol Nutr.

[34] Derks IPM, Bolhuis K, Sijbrands EJG, Gaillard R, Hillegers MHJ, Jansen PW (2019). Predictors and patterns of eating behaviors across childhood: Results from The Generation R study. Appetite.

[35] Yelverton CA, Geraghty AA, O Brien EC, Killeen S, Horan MC, Donnelly JM (2021). Breastfeeding and maternal eating behaviours are associated with child eating behaviours: findings from the ROLO Kids Study. Eur J Clin Nutr.

[36] Herle M, Fildes A, Llewellyn CH (2018). Emotional eating is learned not inherited in children, regardless of obesity risk: Child emotional eating is not heritable. Pediatr Obes.

[37] Shriver LH, Dollar JM, Lawless M, Calkins SD, Keane SP, Shanahan L (2019). Longitudinal associations between emotion regulation and adiposity in late adolescence: indirect effects through eating behaviors. Nutrients.

[38] Delahunt A, Conway MC, McDonnell C, O Reilly SL, O Keeffe LM, Kearney PM (2021). Sleep duration and eating behaviours are associated with body composition in 5-year-old children: findings from the ROLO longitudinal birth cohort study. Br J Nutr.

[39] Lee JS, Jin MH, Lee HJ (2022). Global relationship between parent and child obesity: a systematic review and meta-analysis. Clin Exp Pediatr.

[40] Taylor CM, Steer CD, Hays NP, Emmett PM (2018). Growth and body composition in children who are picky eaters: a longitudinal view. Eur J Clin Nutr.

[41] Herle M, Stavola BD, Hübel C, Abdulkadir M, Ferreira DS, Loos RJF (2020). A longitudinal study of eating behaviours in childhood and later eating disorder behaviours and diagnoses. Br J Psychiatry.

[42] Vilela S, Hetherington MM, Oliveira A, Lopes C (2018). Tracking diet variety in childhood and its association with eating behaviours related to appetite: The generation XXI birth cohort. Appetite.

[43] Goh DY, Jacob A (2012). Perception of picky eating among children in Singapore and its impact on caregivers: a questionnaire survey. Asia Pacific Fam Med.

[44] Sweetman C, Wardle J, Cooke L (2008). Soft drinks and ‘desire to drink’ in preschoolers. Int J Behav Nutr Phys Act.

[45] Domoff SE, Miller AL, Kaciroti N, Lumeng JC (2015). Validation of the Children’s Eating Behaviour Questionnaire in a low-income preschool-aged sample in the United States. Appetite.

[46] Nicklas TA, Morales M, Linares A, Yang S-J, Baranowski T, De Moor C (2004). Children’s meal patterns have changed over a 21-year period: the Bogalusa heart study. Journal of the American Dietetic Association.

[47] Thomson JL, Tussing-Humphreys LM, Goodman MH, Landry AS (2019). Diet quality in a nationally representative sample of American children by sociodemographic characteristics. Am J Clin Nutr.

[48] O’Connor TM, Yang S-J, Nicklas TA (2006). Beverage Intake Among Preschool Children and Its Effect on Weight Status. Pediatrics (Evanston).

