# Supplementary material for: Stability of child appetitive traits and association with diet quality at 5 years and 9–11 years old: Findings from the ROLO longitudinal birth cohort study
Source: Eur J Clin Nutr. 2024 Apr 4;78(7):607–14. doi: 10.1038/s41430-024-01436-6 (PMC11230891; doi:10.1038/s41430-024-01436-6)
Supplement: Supplementary file 1 — Revised Supplementary Tables [file 41430_2024_1436_MOESM1_ESM.docx]

| **Supplementary Table 1:** **Demographic details of children with CEBQ at both timepoints** | | | |
| --- | --- | --- | --- |
|  | **n (%)** | **Mean (Median)** | **SD (IQR)** |
| **Child characteristics at 5yrs old** | | | |
| Child age (years) | 154 | 5.15 | 0.12 |
| Male, n (%) | 75 (44.9) | - | - |
| Breastfeeding exposure | 117 (76.0) | - | - |
| Age of solids introduction (weeks) | 152 | (26) | (20.00,26.00) |
| BMI (kg/m^2^) | 152 | 16.17 | 1.39 |
| BMI z-score | 152 | 0.39 | 0.90 |
| BMI category (overweight/obese) | 37 (24.3) | - | - |
| **Child appetitive traits at 5yrs old** | | | |
| Food Responsiveness | 167 | 2.53 | 0.84 |
| Emotional Overeating | 167 | 1.63 | 0.51 |
| Enjoyment of Food | 167 | 3.79 | 0.72 |
| Desire to Drink | 167 | 2.63 | 0.94 |
| Satiety Responsiveness | 167 | 3.07 | 0.66 |
| Slowness eating | 167 | 3.02 | 0.77 |
| Emotional Undereating | 167 | 2.70 | 0.87 |
| Food Fussiness | 167 | 3.00 | 1.04 |
| **Child characteristics at 9-11yrs old** | | | |
| Child age | 167 | 9.87 | 0.71 |
| Male, n (%) | 75 (44.9) | - | - |
| BMI (kg/m^2^) | 155 | 17.50 | 2.63 |
| BMI z-score | 155 | 0.25 | 1.07 |
| BMI z-score category (overweight/obese) | 39 (25.2) | - | - |
| **Child appetitive traits at 9-11yrs old** | | | |
| Food Responsiveness | 167 | 2.52 | 2.52 |
| Emotional Overeating | 167 | 1.99 | 0.57 |
| Enjoyment of Food | 167 | 3.98 | 0.65 |
| Desire to Drink | 167 | 2.45 | 0.85 |
| Satiety Responsiveness | 167 | 2.71 | 0.66 |
| Slowness eating | 167 | 2.63 | 0.78 |
| Emotional Undereating | 167 | 2.57 | 0.70 |
| Food Fussiness | 167 | 2.81 | 0.96 |
| *Results presented as mean (standard deviation) for normally distributed data, median (25^th^ and 75^th^ percentile) for non-parametric data, and n (%) for categorical data. Abbreviations: RCT; Randomised controlled group, BMI; body mass index.* | | | |

| **Supplementary Table 2:** **Correlation between child appetitive traits and energy intake and Healthy Eating Index at 5 and 9-11 years old** | | | | | | | | |
| --- | --- | --- | --- | --- | --- | --- | --- | --- |
|  | **5 years old** | | | | **9-11 years old** | | | |
|  | **HEI** | | **Energy (kcals)** | | **HEI** | | **Energy (kcal)** | |
|  | **r** | **p-value** | **r** | **p-value** | **r** | **p-value** | **r** | **p-value** |
| **Food Responsiveness** | 0.201 | <0.001 | 0..044 | 0.446 | -0.037 | 0.593 | 0.200 | 0.004 |
| **Emotional Overeating** | 0.156 | 0.006 | 0.071 | 0.218 | -0.072 | 0.304 | 0.193 | 0.005 |
| **Enjoyment of Food** | 0.286 | <0.001 | -0.080 | 0.168 | 0.154 | 0.026 | -0.025 | 0.720 |
| **Desire to Drink** | -0.138 | 0.016 | -0.155 | 0.007 | -0.198 | 0.004 | 0.190 | 0.006 |
| **Satiety Responsiveness** | -0.203 | <0.001 | 0.147 | 0.010 | -0.106 | 0.128 | 0.015 | 0.835 |
| **Slowness Eating** | -0.037 | 0.515 | 0.012 | 0.833 | -0.018 | 0.794 | -0.050 | 0.471 |
| **Emotional Undereating** | 0.091 | 0.113 | 0.166 | 0.004 | -0.015 | 0.828 | 0.092 | 0.185 |
| **Food Fussiness** | -0.345 | <0.001 | 0.063 | 0.275 | -0.347 | <0.001 | 0.152 | 0.029 |
| *Pearson’s correlations; Energy (kcals) log transformed. Statistically significant (p=<0.05)* | | | | | | | | |
